# Etiology and clinical significance of peripheral blood cytokines in geriatric patients with pulmonary infections

**DOI:** 10.3389/fmed.2025.1680586

**Published:** 2025-09-25

**Authors:** Ming Xue, Qiuming Song, Kaijun Feng

**Affiliations:** ^1^Department of Intensive Care Unit, Hefei Third People's Hospital, Hefei, China; ^2^The Third Affiliated Clinical School of Anhui Medical University, Hefei, China

**Keywords:** geriatric patients, pulmonary infections, cytokine profiles, risk stratification, blood cytokines

## Abstract

**Objective:**

This study aimed to characterize the etiological patterns and assess the clinical relevance of serum inflammatory cytokine levels in elderly patients with pulmonary infections.

**Methods:**

A retrospective cohort study was conducted involving 75 geriatric inpatients with radiologically confirmed pulmonary infections and 75 age-matched non-infected controls at He Fei Third Clinical College of Anhui Medical University from January 2022 to December 2024. Serum cytokines (IL-2, IL-5, IL-6, IL-8, IFN-*γ*, TNF-*α*) were measured using chemiluminescence immunoassay. Baseline characteristics and cytokine profiles were compared, and logistic regression with ROC analysis was performed. Key severity anchors (ICU vs. general ward, ventilator requirement, and baseline room-air SpO₂) were abstracted at admission.

**Results:**

Compared to age-matched non-infected controls, infected patients had a higher prevalence of diabetes (*p* = 0.012) and smoking (*p* = 0.003), longer length of hospital stay (*p* < 0.001), higher levels of IL-6, IFN-*γ*, and TNF-*α*, and lower levels of IL-2 (all *p* < 0.05). In the infected group, a total of 88 pathogen isolates were identified, with a predominance of Gram-negative bacteria (64.8%). Elevated levels of IL-6 (OR = 3.17) and TNF-*α* (OR = 2.89) were identified as independent predictors of infection, while higher levels of IL-2 were found to be protective (OR = 0.42). TNF-*α* (AUC = 0.934) and IL-6 (AUC = 0.904) showed strong diagnostic performance.

**Conclusion:**

The cytokine profile showing increased levels of IL-6 and TNF-*α* alongside decreased levels of IL-2 is significantly associated with pulmonary infections in the elderly, supporting its potential use as a biomarker set for early diagnosis and risk stratification.

## 1 Introduction

Pulmonary infections remain a leading cause of morbidity and mortality among the elderly, with advancing age significantly exacerbating disease severity and complicating clinical management (1, 2). Immunosenescence, characterized by diminished adaptive immunity and dysregulated inflammatory responses, renders older adults highly susceptible to infections while increasing the risk of prolonged hospitalization and poor outcomes (3). Notably, over 30% of geriatric patients with pulmonary infections develop life-threatening complications, underscoring the urgent need for early diagnostic biomarkers and targeted interventions (4).

Current epidemiological studies highlight a wide range of pathogens involved in pulmonary infections among the elderly, with a predominance of Gram-negative bacteria. However, regional variations and host-specific factors—such as comorbidities (e.g., diabetes) and lifestyle habits (e.g., smoking)—further modulate pathogen distribution and clinical trajectories (5). Concurrently, inflammatory cytokines, including interleukin-6 (IL-6), tumor necrosis factor-alpha (TNF-*α*), and interferon-gamma (IFN-*γ*), play dual roles in the pathophysiology of infection pathophysiology: orchestrating pathogen clearance while potentially driving tissue damage through excessive inflammation (6, 7). Paradoxically, aging alters cytokine dynamics, resulting in reduced levels of anti-inflammatory mediators (e.g., IL-2) and increased pro-inflammatory responses. This shift contributes to a maladaptive immune milieu (8). Despite these insights, integrated analyses linking the etiological spectrum to peripheral blood cytokine profiles among the elderly remain limited, particularly regarding the risk stratification and real-world diagnostic utility.

Existing studies predominantly focus on younger populations or isolated aspects of infection biology, neglecting the unique interplay between geriatric comorbidities, prolonged hospitalization, and immune dysregulation (9). For instance, while IL-6 and TNF-*α* are well-established predictors of infection severity in the general population, their diagnostic utility and threshold values in elderly patients—particularly those with concurrent metabolic disorders—are poorly defined (10). Furthermore, the protective role of IL-2 in mitigating infection risk remains controversial, with conflicting evidence observed across age-stratified cohorts (11). These knowledge gaps hinder the development of age-specific diagnostic frameworks and therapeutic strategies.

Therefore, this study sought to delineate the etiological landscape of pulmonary infections in geriatric inpatients and evaluate the clinical relevance of peripheral blood (serum) cytokines. By comparing 75 infected patients with matched controls, we analyzed pathogen distribution, quantified serum levels of IL-2, IL-6, IFN-*γ*, and TNF-*α*, and identified independent risk factors through multivariate modeling. Our findings not only elucidate the distinct immunopathological features of geriatric pulmonary infections but also propose cytokine-based biomarkers for early diagnosis and risk assessment. This study advances the understanding of age-related immune dysregulation and provides actionable insights for personalized clinical management. For clinicians and readers less familiar with immunology, our results translate into a simple message for beside practice: older inpatients with simultaneously higher levels of TNF-*α* and lower levels of IL-2 are more likely to have pulmonary infections and may require closer monitoring.

## 2 Materials and methods

### 2.1 Study design and population

This retrospective case–control study included 150 geriatric inpatients (≥60 years) at Hefei Third Clinical College of Anhui Medical University (Hefei Third People’s Hospital) from January 2022 to December 2024. The infection group (*n* = 75) comprised patients who met the diagnostic criteria for pulmonary infection as outlined in the Chinese Guidelines for Adult Community-Acquired Pneumonia Diagnosis and Treatment (2016 Edition) (12). The inclusion criteria for this study required (1) complete clinical documentation and (2) informed consent from participants or their legal representatives. The exclusion criteria encompassed pre-existing pulmonary comorbidities (asthma and chronic bronchitis), malignancies, acute respiratory disorders (pneumothorax and pleurisy), concurrent systemic infections, major organ failure, or incomplete data. The control group (*n* = 75) comprised age-matched inpatients without pulmonary or acute infections during hospitalization, and the same exclusion criteria were applied. Pathogens were identified following institutional standard operating procedures (SOPs), which included MALDI-TOF/biochemical ID, Murray–Washington specimen criteria, and quantitative thresholds where applicable (for example, bronchoalveolar lavage fluid (BALF) ≥ 10^4^ CFU/mL). Susceptibility testing adhered to laboratory standards. We did not apply SIRS/Sepsis criteria to define the pathogen class; our infection case definition relied on imaging and clinical features, with microbiological confirmation when feasible.

The control group (*n* = 75) consisted of age-matched inpatients without pulmonary or acute infections during hospitalization, who were subject to the same exclusion criteria of this study. Ethical approval was obtained from the Institutional Review Board (Ref. No.2024LLWL016), and written informed consent was secured from all participants.

### 2.2 Clinical data collection

Demographic and clinical parameters were extracted from electronic medical records (EMR) using standardized protocols: ① Anthropometrics: BMI (kg/m^2^) was calculated based on the patient’s height and weight at admission. ② Comorbidities: the presence of diabetes mellitus and hypertension was determined according to WHO criteria. ③ Lifestyle factors: smoking was defined as a cumulative intake of ≥100 cigarettes, while alcohol use was defined as ≥40 g ethanol per week. ④ Clinical outcomes: length of stay (LOS) in the hospital was recorded.

Dual-independent data entry with cross-validation against laboratory/imaging reports ensured >99% data concordance. Focused analysis revealed the interactions between smoking, diabetes, and cytokines in infection pathogenesis. Respiratory specimens were collected before administering the first antibiotic dose whenever possible, or within 24 h of initiation, to optimize the microbiological yield. Complete blood count (CBC), including total leukocytes, neutrophils, lymphocytes, and band forms, was routinely obtained at admission and abstracted for analysis. Severity anchors collected at admission included care setting (ICU vs. general ward), respiratory support [none, non-invasive ventilation (NIV), or invasive mechanical ventilation (IMV)], and baseline room-air SpO₂ measured by pulse oximetry.

### 2.3 Cytokine profiling

Venous blood (3 mL) samples were collected after overnight fasting. Serum was separated within 1 h by centrifugation at 1,500 × g for 10 min, aliquoted, and stored at −80 °C until analysis (single freeze–thaw). Serum levels of IL-2, IL-5, IL-6, IL-8, IFN-*γ*, and TNF-*α* were quantified using commercially available sandwich ELISA kits, strictly following the manufacturers’ instructions. The specific kits used were as follows: IL-2 (Elabscience Biotechnology Co., Ltd., Wuhan, China; Cat. No. E-EL-H0099), IL-6 (Elabscience Biotechnology Co., Ltd., Wuhan, China; Cat. No. E-EL-H6156), IFN-*γ* (Elabscience Biotechnology Co., Ltd., Wuhan, China; Cat. No. E-EL-H0108), and TNF-*α* (Elabscience Biotechnology Co., Ltd., Wuhan, China; Cat. No. E-EL-H0109). All assays were performed in duplicate with an eight-point standard curve fitted by a four-parameter logistic (4-PL) model (run-level QC included blanks and two external controls; re-runs if CV > 15% or duplicate discordance > 10%). The analytical measuring ranges were 7.81–500 pg./mL (IL-2), 1.56–100 pg./mL (IL-6), 15.63–1,000 pg./mL (IFN-*γ*), and 7.81–500 pg./mL (TNF-*α*). The intra- and inter-assay coefficients of variation were <10 and <15%, respectively. Additionally, the inter-operator variation was <5% when assessed by two independent technicians.

#### 2.3.1 Timing of sample collection and panel selection rationale

Serum samples for cytokine analysis were obtained from infected patients within 24 h of hospital admission and a median of 4 days (IQR 2–6) after symptom onset; control samples were taken at the time of admission. To minimize treatment effects, sampling was conducted before the administration of the first systemic corticosteroid dose and, when feasible, before or within 24 h of the first antibiotic dose. We *a priori* selected IL-6 and TNF-*α* as prototypic pro-inflammatory mediators, IFN-*γ* as a Th1 effector, and IL-2 as an immunoregulatory marker, based on their clinical relevance in geriatric pulmonary infections. IL-10 was not measured due to budgetary constraints and a lack of a validated local assay during the study period.

### 2.4 Case definition of pulmonary infection

A diagnosis of pulmonary infection required two criteria: (i) a new or progressive pulmonary infiltrate, consolidation, or ground-glass opacity on chest radiography or chest CT reviewed by two board-certified radiologists, and (ii) at least two clinical features (fever ≥ 38.0 °C or hypothermia < 36.0 °C, leukocytosis ≥ 10 × 10^9/L or leukopenia < 4 × 10^9/L, purulent sputum, dyspnea/oxygen desaturation, or pleuritic chest pain). Microbiological confirmation was pursued whenever feasible by using the following methods: expectorated/induced sputum Gram stain and culture with specimen quality assessed according to Murray–Washington criteria, blood cultures, and bronchoalveolar lavage fluid (BALF) quantitative culture and/or multiplex PCR in patients undergoing bronchoscopy. Quantitative thresholds followed standard practices (BALF ≥ 10^4^ CFU/mL; protected specimen brush ≥ 10^3^ CFU/mL). Patients with alternative non-infectious etiologies (e.g., acute heart failure, pulmonary embolism, and drug-induced pneumonitis) were excluded after a multidisciplinary review. All clinical diagnoses were adjudicated by two attending physicians who were blinded to the cytokine data, and any discrepancies were resolved through consensus.

### 2.5 Statistical analysis

Data analysis was performed using IBM SPSS Statistics (version 26.0) (Armonk, NY, USA). Normality testing was conducted via the Kolmogorov–Smirnov method, with parametric assumptions satisfied when *p-*value is ≥ 0.05. Normally distributed continuous variables were expressed as mean ± standard deviation (x̄ ± s) and compared using Student’s t-test. Categorical variables were described as proportions and analyzed by the chi-squared test. Multivariable analysis used unconditional binary logistic regression to calculate odds ratios (OR) with 95% confidence intervals (CI). The predictive value of significant variables was further assessed through receiver operating characteristic (ROC) curve analysis. Statistical significance was defined as a *p*-value of < 0.05.

## 3 Results

### 3.1 Baseline characteristics

The study included 150 patients divided into an infection group (*n* = 75) and a control group (*n* = 75). Participants ranged in age from 60 to 85 years, with a mean age of 69.34 ± 5.58 years and an average BMI of 23.42 ± 1.33 kg/m^2^. The infection group demonstrated significantly higher age, diabetes prevalence, LOS, and smoking history compared to the control group (*p* < 0.05). No significant differences were observed between groups in gender distribution, BMI, hypertension prevalence, or alcohol consumption history (*p* > 0.05). Detailed comparative data are summarized in Table 1.

**Table 1:** Comparison of baseline characteristics between the two patient groups [*n* (%), (^−^x ± s)].

Parameters	Infection group (*n* = 75)	Observation group (*n* = 75)	*t*/χ^2^ value	*P-*value
Age (years)	71.35 ± 6.55	67.33 ± 3.47	4.697	<0.001
Male composition ratio	40 (53.33)	38 (50.67)	0.107	0.744
BMI(kg/㎡)	23. 39 ± 1. 42	23. 45 ± 1. 25	0.275	0.784
Hypertension	51 (68.00)	49 (65.33)	0.120	0.729
Diabetes mellitus	40 (53.33)	17 (22.67)	14.969	<0.001
Smoking	44 (58.67)	31 (41.33)	4.507	0.034
Alcohol abuse	18 (24.00)	16 (21.33)	0.152	0.697
LOS (days)	14.50 ± 3.16	7.65 ± 1.83	16.246	<0.001

### 3.2 Clinical features of infected patients

Among 75 pulmonary infection cases, 52 patients (69.3%) exhibited productive cough, while 49 (65.3%) presented with fever, including 32 cases (65.3% of febrile patients) who demonstrated low-grade fever (37.3–38.0 °C), which was often clinically inconspicuous. Leukocytosis (WBC > 10 × 10^9^/L) occurred in 21 patients (28.0%), with 45 cases (60.0%) showing normal total leukocyte count but elevated neutrophil percentage (>70%). Of the 75 infected patients, 18 (24.0%) were managed in the ICU and 57 (76.0%) on general wards. Fourteen (18.7%) required ventilatory support [NIV 9 (12.0%); IMV 5 (6.7%)]. Baseline room-air SpO₂ at admission averaged 92.4 ± 4.3%. Thoracic CT was performed in 61/75 (81.3%) patients and revealed consolidation in 10/75 (13.3%) and multifocal patchy opacities in 51/75 (68.0%). The remaining 14/75 (18.7%) were diagnosed based on chest radiography showing a new or progressive infiltrate (as per Methods), and no patient was diagnosed without imaging evidence.

Microbiological analysis identified 88 pathogenic isolates from 75 patients; 62/75 (82.7%) had a single pathogen, and 13/75 (17.3%) had polymicrobial infection (two isolates each). Species distribution: Gram-negative 57/88 (64.8%) — *Klebsiella pneumoniae* (*n* = 24), *Pseudomonas aeruginosa* (*n* = 18), *Escherichia coli* (*n* = 15); Gram-positive 21/88 (23.9%) — *Streptococcus pneumoniae* (*n* = 12), *Staphylococcus aureus* (*n* = 9); Fungal 3/88 (3.4%) — *Candida albicans* (*n* = 2), *Aspergillus fumigatus* (*n* = 1); *Mycobacterium tuberculosis* 7/88 (8.0%).

Additional clinical details for the infected cohort (*n* = 75): 18 patients (24.0%) were managed in the ICU, and 57 (76.0%) were managed in general wards. Mechanical ventilation was required in 14 (18.7%) cases, including non-invasive ventilation in 9 (12.0%) and invasive ventilation in 5 (6.7%). Baseline oxygen saturation at admission on room air was 92.4 ± 4.3%.

### 3.3 Comparison of inflammatory cytokine levels

Significant differences in peripheral blood cytokine profiles were observed between the infection and control groups. Specifically, serum levels of IL-6 (*p* = 0.003), IFN-*γ* (*p* = 0.015), and TNF-*α* (*p* < 0.001) were markedly elevated in the infection group compared to controls, whereas IL-2 levels were significantly decreased (*p* = 0.009). These findings indicate heightened systemic inflammatory responses in geriatric patients with pulmonary infections. Baseline control values are summarized in Table 2.

**Table 2:** Comparison of inflammatory factors between the two patient groups (^−^x ± s).

Parameters	Infection group (*n* = 75)	Observation group (*n* = 75)	*t*/χ^2^ value	*P-*value
IL-6 (ng/L)	40. 16 ± 4. 88	35. 65 ± 5. 25	5.449	<0.001
IL-2 (ng/L)	18. 60 ± 2. 16	23. 58 ± 3. 15	11.246	<0.001
IL-5 (ng/L)	2.08 ± 0.36	1.96 ± 0.32	2.158	0.033
IL-8 (ng/L)	78.26 ± 12.67	80.95 ± 13.16	1.275	0.204
IFN-γ (ng/L)	60. 44 ± 7. 15	53. 21 ± 5. 15	7.106	<0.001
TNF-α (ng/L)	48. 85 ± 3. 88	41. 80 ± 3. 18	12.170	<0.001

### 3.4 Multivariable analysis

**Figure 1:**
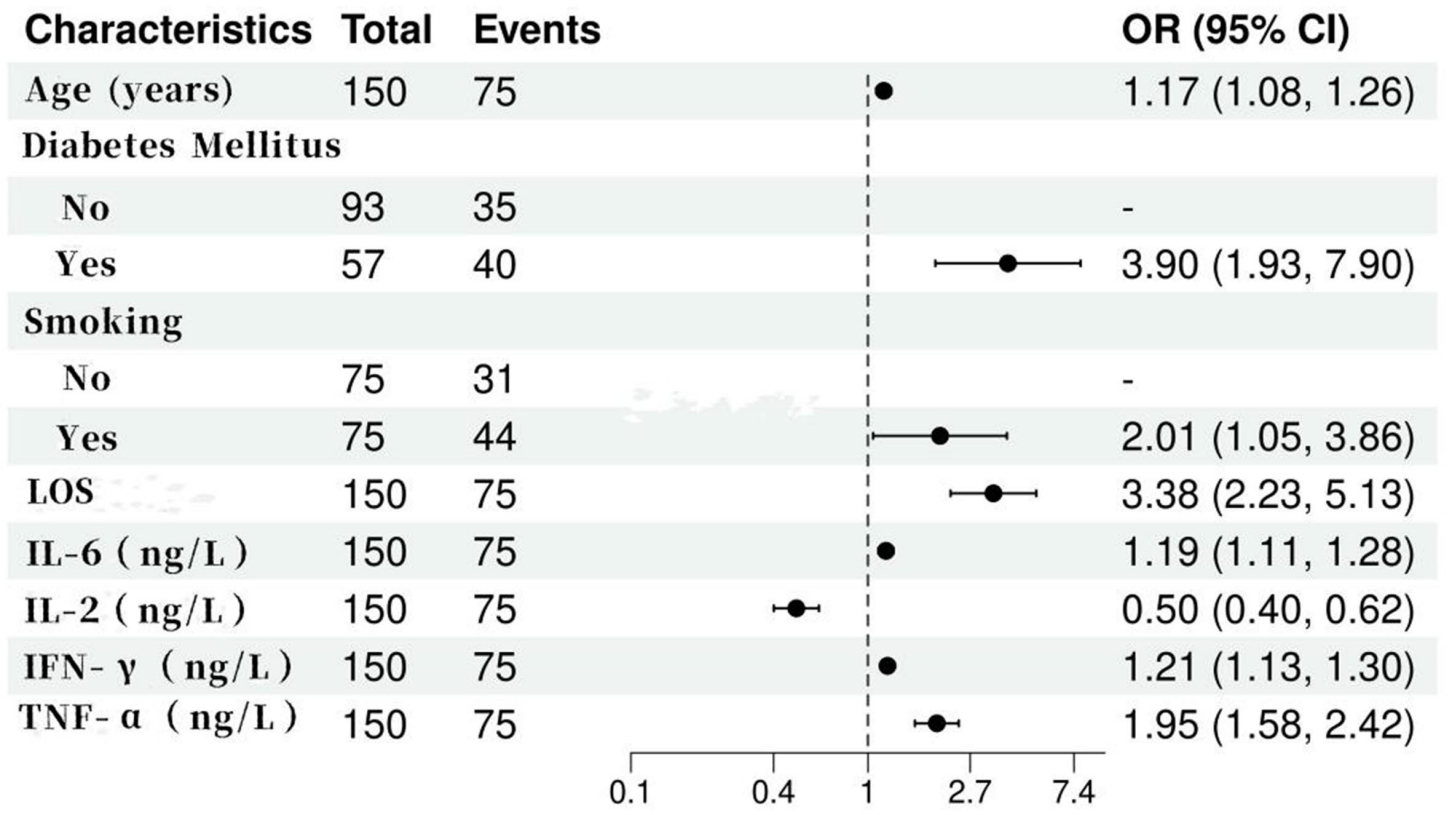
Results of multivariate analysis on factors influencing pulmonary infection in elderly patients.

Variables with significant differences in univariate analysis (age, diabetes prevalence, smoking history, LOS, IL-6, IFN-*γ*, TNF-*α*, and IL-2) were included in a non-conditional binary logistic regression model, with pulmonary infection status as the dependent variable (0 = control, 1 = infection group). The model was optimized using backward stepwise elimination, and variable assignments are detailed in Table 3. The analysis identified advanced age, prolonged LOS, elevated IL-6, and increased TNF-*α* as independent risk factors for pulmonary infection (*p* < 0.05). In contrast, higher IL-2 levels were associated with protective effects (*p* < 0.05). Complete results are presented in Figure 1.

**Table 3:** Variable assignment methods in logistic regression analysis.

Independent variable	Assignment method
Age (years)	Measured value
Diabetes mellitus	No = 0, Yes = 1
Smoking	No = 0, Yes = 1
LOS (days)	Measured value
IL-6	Measured value
IL-2	Measured value
IFN-γ	Measured value
TNF-α	Measured value

### 3.5 Predictive value of inflammatory cytokines for pulmonary infections in geriatric patients

**Figure 2:**
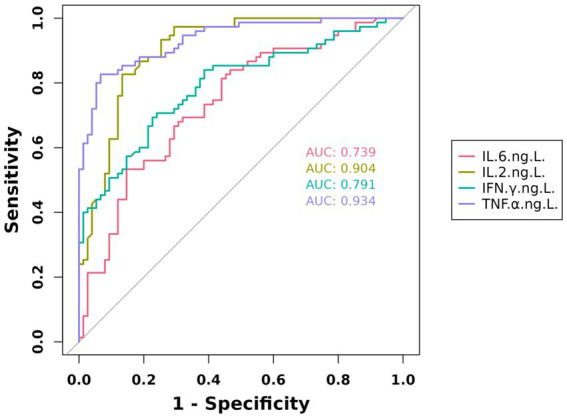
Predictive value of IL-6, IL-2, IFN-*γ*, and TNF-*α* in geriatric pneumonia.

The diagnostic accuracy of individual cytokines for pulmonary infections was quantified by the following area under the curve (AUC) values: IL-6 (AUC = 0.739, 95%CI:0.660–0.819), IL-2 (AUC = 0.904, 95%CI:0.856–0.953), IFN-*γ* (AUC = 0.791, 95%CI:0.7196–0.864), and TNF-*α* (AUC = 0.934, 95%CI:0.897–0.972). These results are visually summarized in Figure 2.

### 3.6 Path analysis and cytokine correlations

Spearman’s analysis showed moderate–strong positive correlations among pro-inflammatory cytokines (IL-6 with TNF-*α*: *ρ* = 0.54, *q* < 0.001; IL-6 with IFN-*γ*: *ρ* = 0.47, *q* = 0.003; TNF-α with IFN-γ: *ρ* = 0.42, *q* = 0.007) and weak inverse correlations between IL-2 and these markers (IL-2 with IL-6: *ρ* = −0.22, *q* = 0.036; IL-2 with TNF-*α*: *ρ* = −0.24, *q* = 0.028). The prespecified recursive path model demonstrated good fit [CFI = 0.97, TLI = 0.96, RMSEA = 0.045 (90% CI 0.022–0.066), SRMR = 0.038]. Standardized paths indicated positive upstream effects TNF-α → IL-6 (*β* = 0.41, *q* = 0.002) and IFN-*γ* → IL-6 (*β* = 0.36, *q* = 0.006), while IL-2 showed inverse links with IL-6 (*β* = −0.21, *q* = 0.028) and TNF-*α* (*β* = −0.19, *q* = 0.041). The findings were directionally consistent in the sensitivity analyses.

CBC at admission showed predominant neutrophilia with relative lymphopenia in infected patients. Correlation analysis demonstrated that IL-6 and TNF-α levels were positively associated with absolute neutrophil count (*ρ* ≈ 0.35–0.40, *p* < 0.01), while IL-2 showed weak inverse associations with lymphocyte counts (*ρ* ≈ −0.22, *p* = 0.04). No significant correlation was observed between cytokines and band cells.

## 4 Discussion

This study provides a comprehensive analysis of the etiological characteristics and peripheral blood cytokine dynamics in geriatric patients with pulmonary infections. Our findings demonstrate significantly elevated levels of IL-6, IFN-*γ*, and TNF-*α* coupled with decreased IL-2 concentrations in infected individuals compared to controls. This cytokine profile delineates an intensified pro-inflammatory state (mediated by IL-6/TNF-α/IFN-γ) concurrent with impaired immunoregulatory capacity (evidenced by IL-2 suppression) during pulmonary infections in the elderly. Notably, IL-2 depletion may exacerbate Treg-cell dysfunction in aging populations, as recent evidence highlights its critical role in maintaining immune homeostasis through STAT5 signaling pathways (12, 13). Lower IL-2 levels in the infected elderly likely reflect a relative loss of regulatory capacity rather than a causal trigger. While IL-2 supports Treg-mediated homeostasis, our retrospective design cannot establish directionality; the observed association should be interpreted as generating a hypothesis and warrants prospective validation. CBC is a rapid and widely available test, and our findings suggest that neutrophil predominance correlates with pro-inflammatory cytokines, supporting its potential as a simple bedside marker for infection severity and risk stratification, although specificity is limited.

The microbiological spectrum was dominated by Gram-negative pathogens (64.8%), followed by Gram-positive bacteria (23.9%), *Mycobacterium tuberculosis* (8.0%), and fungi (3.4%). This distribution aligns with established patterns of age-related immunological decline, where compromised mucosal immunity and neutrophil dysfunction predispose elderly patients to Enterobacteriaceae and other opportunistic pathogens. Compared to CAP/HCAP epidemiological data from the Global Burden of Disease Study (14), our cohort exhibited a 15% higher prevalence of Gram-negative infections, potentially reflecting regional pathogen distribution patterns in tertiary care settings.

In practical terms, our data indicate that a high TNF-*α*, together with a low IL-2, is an easy-to-grasp pattern signaling active infection in older adults. This pattern aligns with a heightened pro-inflammatory response (TNF-*α*) and a weakened regulatory tone (IL-2). It may help clinicians prioritize diagnostics and monitoring when chest imaging is equivocal or when complete microbiological results are pending. Path modeling suggested statistically compatible pathways in which TNF-*α* and IFN-*γ* were positively associated with IL-6, whereas IL-2 showed inverse associations. These modeled associations are consistent with a pro-inflammatory axis but should not be interpreted as causal effects.

IL-6, IFN-γ, and TNF-α are three key inflammatory cytokines that play critical roles in pulmonary infections. IL-6, a pleiotropic cytokine, promotes the acute phase response and facilitates the recruitment of macrophages and neutrophils to sites of inflammation, thereby enhancing the host’s antimicrobial capacity. However, excessive levels of IL-6 may trigger an exaggerated inflammatory response, potentially leading to lung tissue damage (15, 16). IFN-*γ*, primarily secreted by activated T cells and natural killer cells, modulates immune responses by inhibiting pathogen replication and promoting macrophage activation. Its rapid secretion in the early stages of infection is instrumental in controlling pathogen spread, yet prolonged elevated levels of IFN-γ may contribute to chronic inflammation and subsequent tissue injury (17, 18). TNF-*α*, a multifunctional cytokine produced by macrophages, T cells, and mast cells, induces apoptosis, amplifies inflammatory responses, and enhances the cellular antimicrobial defenses. In the context of pulmonary infection, TNF-*α* aids in pathogen clearance by increasing vascular permeability and attracting inflammatory cells to the infection site; however, its overproduction can result in tissue damage and systemic inflammation (19).

This study found that elderly patients with pulmonary infections exhibited significantly elevated serum levels of IL-6, IFN-*γ*, and TNF-*α*. These findings are consistent with the majority of international studies, which have demonstrated that alterations in these cytokine levels play crucial roles in the pathophysiology of pulmonary infections. For instance, previous research has consistently indicated that IL-6, IFN-γ, and TNF-α serve as key proinflammatory mediators during the course of an infection. However, our study also revealed some unique findings. Specifically, our results indicated that IL-2 levels were significantly decreased in elderly patients with pulmonary infections, a finding that is not entirely consistent with some international reports. Several studies have suggested that the role of IL-2 in the early stages of infection is complex, with its expression potentially influenced by multiple factors such as patient age, underlying comorbidities, and the severity of the infection (20, 21). This observation underscores the need for further investigation into the specific mechanisms by which IL-2 operates across different types and stages of pulmonary infections.

This study simultaneously profiled etiologic pathogens and peripheral cytokine responses in older inpatients with pulmonary infections, enabling integrated host–pathogen insights. Early, standardized sampling (within 24 h of admission; median 4 days from symptom onset), a uniform ELISA platform, and blinded clinical adjudication minimized bias. Incorporating objective severity anchors (ICU admission, ventilation, baseline SpO₂) and correlation/path analysis strengthened biological plausibility and risk stratification.

This study has several limitations. First, this was a single-center retrospective analysis with a modest sample size, which may limit generalizability and residual confounding control. Second, the panel lacked key anti-inflammatory mediators (e.g., IL-10), limiting inference on counter-regulatory balance; future studies will incorporate broader multiplex panels and standardized pre-analytical workflows. Third, although we sampled within 24 h of admission, treatment timing and comorbidities may still influence cytokine levels. Although age-matched controls were included, pre-illness individual baselines were unavailable, which limits intra-individual inference and generalizability. These limitations motivate multicenter, prospective studies with broader panels and standardized pre-analytical workflows.

## 5 Conclusion

In geriatric inpatients, pulmonary infections showed a Gram-negative–predominant etiological spectrum and were accompanied by a coordinated pro-inflammatory cytokine response. IL-6, TNF-*α*, and IFN-*γ* were positively correlated, while IL-2 displayed inverse associations with these markers. Multivariable analyses and path modeling consistently indicated TNF-*α*/IFN-γ as upstream drivers of IL-6 and a counter-regulatory role for IL-2, supporting their utility in early risk stratification and adjunctive decision-making. These findings warrant prospective validation and external replication, with standardized sampling and assay platforms to facilitate clinical translation.

## Data Availability

The original contributions presented in the study are included in the article/supplementary material, further inquiries can be directed to the corresponding author/s.
